# Strontium‐Containing Bioactive Glass Nanoparticles Stimulate Osteogenesis and Suppress Osteoclast Formation in Co‐Culture

**DOI:** 10.1002/adhm.202503671

**Published:** 2025-10-24

**Authors:** Parichart Naruphontjirakul, Alexandra E. Porter, Julian R. Jones

**Affiliations:** ^1^ Biological Engineering Program Faculty of Engineering King Mongkut's University of Technology Thonburi Bangkok 10140 Thailand; ^2^ Department of Materials Imperial College London South Kensington Campus London SW7 2AZ UK

**Keywords:** anti‐osteoclastic activity, bioactive glass, indirect co‐culture system, nanoparticles, osteoblast activity, strontium

## Abstract

The effect of monodispersed bioactive glass nanoparticles containing strontium (9.4 mol % SrO and 4.4 mol% SrO) and their dissolution products on osteoclast differentiation are investigated in monoculture and in an osteoblast‐osteoclast in vitro co‐culture system. Under standard conditions, RAW264.7 cells efficiently differentiate into osteoclast‐like cells upon RANKL stimulation, making them a well‐established monoculture model for osteoclastogenesis. The viability of RAW264.7 cells exposed to nanoparticles is dose‐ and time‐dependent. Indirect co‐culture of pre‐osteoblast MC3T3‐E1 and mouse monocyte RAW264.7 cells is carried out using Millipore cell culture plate inserts, with and without RANKL. Osteoclast differentiation, indicated by tartrate‐resistant acid phosphatase (TRAP) activity and the formation of multinucleated osteoclasts, is significantly reduced in RAW264.7 cells exposed to Sr‐BGNPs and their dissolution products, compared to RANKL‐stimulated control cells. Therefore, the observed reduction in osteoclast differentiation following exposure to Sr‐BGNPs and their ionic dissolution products indicates a potential inhibitory effect on this process. In co‐culture, bioactive glass nanoparticles containing 9.4 mol% SrO_2_ promoted differentiation of osteoblasts, relative to basal media and osteogenic media controls, measured as an increase in Alkaline phosphotase (ALP) activity and formation of mineralized matrix, while differentiation of osteoclasts decreased, measured as a reduction in TRAP activity and multinucleated osteoclast formation.

## Graphical abstract

Spherical bioactive glass nanoparticles containing strontium inhibit osteoclast differentiation and activity, but promote osteoblast activity in osteoblast‐osteoclast in vitro co‐culture. The nanoparticles could be used for the treatment of osteoporosis.

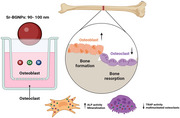

## 1 Introduction

The balance between bone formation and resorption is essential for maintaining skeletal integrity. Osteoblasts, responsible for bone matrix synthesis and mineralization, and osteoclasts, which mediate bone resorption, are dynamically regulated through complex signaling pathways and cellular interactions.^[^ ^1^ ^]^ Osteoblast‐osteoclast communication is a critical factor for bone mass balance and remodeling.^[^ ^2^ ^]^ An imbalance of osteoblast and osteoclast activities leads to many bone disorders, such as osteoporosis.^[^ ^3^ ^]^ Osteoclasts are differentiated cells formed by the fusion of mononuclear precursor cells of the monocyte‐macrophage lineage.^[^ ^4^ ^]^ One of the key regulators of osteoclast differentiation is the receptor activator of nuclear factor kappa‐B ligand (RANKL), a cytokine primarily produced by osteoblasts and bone marrow stromal cells.^[^ ^5^ ^]^ RANKL binds to its receptor RANK on osteoclast precursors, promoting their differentiation into mature osteoclasts.^[^ ^6^ ^]^ Osteoclast differentiation is initiated when pre‐osteoclasts express tartrate‐resistant acid phosphatase (TRAP), followed by exposure to receptor activator of RANKL, leading to multinucleated osteoclast formation.^[^ ^7^ ^]^ It has been reported that the interaction between RANK on osteoclast precursors and its ligand RANKL on osteoblasts or stromal cells plays a critical role in osteoclastogenesis.^[^ ^8^ ^]^ In pathological conditions such as osteoporosis, this balance is disrupted, leading to excessive bone resorption and compromised bone strength. Therefore, the development of biomaterials that can simultaneously stimulate osteoblast activity and suppress osteoclast formation is of considerable interest in regenerative medicine and orthopedic applications.

Bioactive glasses (BGs) are well‐established materials in bone tissue engineering due to their ability to bond with bone through the formation of a hydroxyl carbonate apatite (HCA) layer.^[^ ^9^, ^10^ ^]^ They have a second mode of bioactivity in that their dissolution products have been shown to promote osteoblasts to produce more bone in vitro.^[^ ^11^, ^12^ ^]^ Bioactive glass nanoparticles (BGNPs) have the potential to improve the delivery of active ions for specific applications. BGNPs can be produced through various fabrication techniques, including sol‐gel,^[^ ^13^ ^]^ microemulsion, and flame spraying.^[^ ^14^, ^15^, ^16^ ^]^ Sol‐gel modified Stöber process can control the size and homogeneity of the particles,^[^ ^13^, ^15^, ^17^ ^]^ wherein the pH value is much higher than the isoelectric point (pI) of soluble silica (silicic acid), causing repulsion between newly formed primary particles in the sol. This forms discrete particles in the hydrolysis reaction and termination of condensation, resulting in the formation of spherical secondary particles.^[^ ^18^ ^]^ Cation incorporation can be challenging, and introducing calcium into the composition did not promote human bone marrow stem cell differentiation when the cells were cultured with SiO_2_‐CaO nanoparticles.^[^ ^19^ ^]^

Among other therapeutic ions incorporated into BGs, strontium (Sr^2^⁺) has gained particular attention for its dual action, enhancing osteoblastic function while inhibiting osteoclastic activity.^[^ ^20^, ^21^ ^]^ These effects are thought to mimic the clinical benefits of strontium ranelate (SrR), a drug previously used for osteoporosis treatment. SrR has been shown to stimulate osteoblast differentiation and bone formation, while inhibiting osteoclast differentiation and bone resorption, making it an effective dual‐action agent for the treatment of osteoporosis. However, concerns over its association with an increased risk of myocardial infarction led to restrictions on its clinical use.^[^ ^22^ ^]^ To overcome this, Sr has been substituted for Ca in BGs to deliver the bioactive Sr ions to osteoporotic bone tissue locally, to reduce side effects.^[^ ^20^, ^23^, ^24^ ^]^ The integration of Sr into BGNPs further enhances their bioavailability and facilitates cellular uptake, offering a promising strategy for local, sustained therapeutic effects.^[^ ^20^, ^21^ ^]^

Our previous studies reported that Sr‐BGNPs and their dissolution products were not significantly toxic to MC3T3‐E1 cells or to hMSCs and stimulated osteogenic differentiation in MC3T3‐E1 mono‐cultures ^[^ ^25^, ^26^ ^]^ and unpolarized macrophages (Mo) isolated from female Bal/c mice murine bone marrow.^[^ ^27^ ^]^ These particles also stimulated osteogenic differentiation in hMSC mono‐culture.^[^ ^25^, ^26^ ^]^ Sr plays a role in both stimulating osteoblast activity and inhibiting osteoclastogenesis.

The RAW264.7 cell line is an osteoclast‐like monocyte/macrophage precursor, which is frequently used as an osteoclast model.^[^ ^28^ ^]^ RANKL is a major osteoclastogenic molecule that is commonly used to stimulate osteoclast differentiation in in vitro cell culture by up‐regulating the expression of osteoclast‐associated genes.^[^ ^29^ ^]^ This molecule is used to induce differentiation of RAW264.7 cells, resulting in the fusion of mononuclear cells to form multinucleated osteoclasts.^[^ ^28^, ^30^ ^]^ The formation of multinucleated osteoclasts is a critical step in osteoclast differentiation. TRAP is one of the osteoclastic markers used to investigate the phenotype of multinucleated osteoclasts and is expressed during the differentiation of RAW264.7 cells to osteoclasts.^[^ ^31^, ^32^ ^]^

This study aims to investigate the effect of Sr‐BGNPs and their dissolution ions on the differentiation of monocytes to osteoclast‐like cells in a monoculture and then in co‐culture with pre‐osteoblasts. The first objective was to examine the effects of Sr‐BGNPs and their dissolution products on the differentiation and maturation of RAW264.7 into multinucleated osteoclasts through osteoclast cell viability and TRAP activity, which measure osteoclast phenotypic markers. Then, the differentiation of pre‐osteoblasts to osteoblasts and monocytes to osteoclast‐like cells induced by Sr‐BGNPs and their dissolution products was investigated, in an indirect osteoblast‐osteoclast co‐culture system, to compare the effect of cross‐communication between osteoblasts and osteoclasts. This was done by investigating how adding additional RANKL to the co‐culture with pre‐osteoblast cells, which that should produce RANKL during culture, affects cellular response.

## 2 Experimental Section

Reagents were purchased from Sigma–Aldrich unless stated otherwise. Monodisperse strontium containing bioactive glass nanoparticles with a diameter of ≈100 nm were synthesised using the modified Stöber process described previously, with ∼5 and 10 mol % SrO.^[^ ^26^ ^]^ 32.92 mL of ethyl alcohol (200 proof, 99.5%), 4.11 mL of distilled water, and 0.48 mL of ammonium hydroxide (28.0‐30.0% NH_4_OH basis) were added to a 50 mL centrifuge tube and completely mixed solution in an ultrasonication bath for 10–15 min. Then, 2.50 mL of tetraethyl orthosilicate (TEOS, reagent grade, 98%) was gently dropped to the mixed solution. TEOS was left under ultrasonication for 6 h to complete hydrolysis and polycondensation reactions to form the silica network (Si‐O‐Si). Silica nanoparticles (Si‐NPs) were formed first, before Ca and Sr were incorporated through the addition of calcium nitrate tetrahydrate (99%) and strontium nitrate tetrahydrate (99%). The particles were collected through centrifugation and subsequently dried at 80 °C overnight. Calcination was then carried out at 680 °C to incorporate Ca and Sr and to remove the nitrate byproducts of the precursors. Monodispersed spherical particles with a diameter of ≈90–100 nm are shown in **Table** **1**. The compositions of the particles, measured by inductive coupled plasma optical emission spectroscopy (ICP‐OES) of a lithium metaborate fusion,^[^ ^33^ ^]^ were: 90.6 mol% SiO_2_, 5.0 mol% CaO, 4.4 mol% SrO (termed 4.4Sr‐BGNPs), and 88.8 mol% SiO_2_, 1.8 mol% CaO, 9.4 mol% SrO (termed 9.4Sr‐BGNPs), as reported in our previous work.^[^ ^26^ ^]^ The size and morphology of the synthesized particles were evaluated by dynamic light scattering (DLS, Malvern instrument 2000) and Transmission Electron Microscopy (TEM, JEOL 2100 Plus microscope operated at 200 kV).

**Table 1:** Morphology (TEM) and modal diameter from dynamic light scattering measurements (DLS) of 4.4Sr‐BGNPs and 9.4Sr‐BGNPs, and their composition measured by ICP following lithium metaborate fusion.

Samples	TEM	Modal diameter, DLS [nm]	% mol		
			Si	Ca	Sr
4.4Sr‐BGNPs	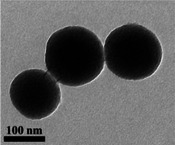	91.3 ± 9.67	90.6 ± 0.16	5.0 ± 0.13	4.4 ± 0.03
9.4Sr‐BGNPs	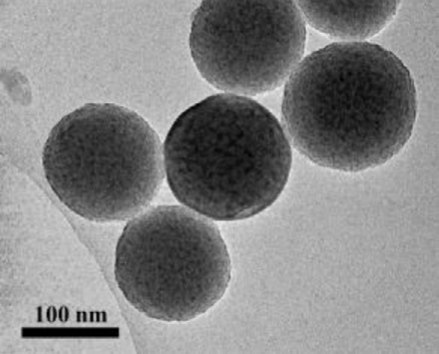	98.7 ± 9.19	88.8 ± 0.30	1.8 ± 0.23	9.4 ± 0.07

### 2.1 Cell Culture

MC3T3‐E1 cells (ATCC CRL‐2593™) were cultured (cell density at 5×10^4^ cells mL^−1^) in T‐75 cell culture flasks (Corning; Sigma–Aldrich) in the basal condition: α‐minimum essential medium (α‐MEM) supplemented with 10% (v/v) fetal bovine serum (FBS), 100 U mL^−1^ penicillin and 100 µg mL^−1^ streptomycin (Gibco; Thermo Fisher Scientific), at 37 °C, 5% CO_2_ and in a fully humidified atmosphere. The cells were detached with trypsin ethylenediaminetetraacetic acid (EDTA) (Gibco; Thermo Fisher Scientific) and sub‐cultured when the cells reached 75–90% confluency,^[^ ^25^ ^]^ ready for co‐culture. Cells were routinely cultured in the basal α‐MEM. Media was replaced twice weekly.

RAW264.7 cells (ATCC TIB‐71^™^) of the mouse leukemic monocyte macrophage cell line obtained from American Type Culture Collection (ATCC), were cultured in Dulbecco's modified Eagle's medium (DMEM) supplemented with 10% (v/v) FBS, 100 U mL^−1^ penicillin, and 100 µg mL^−1^ streptomycin, at 37 °C, 5% CO_2,_ and a fully humidified atmosphere. The culture medium was replaced twice a week, and cells were passaged at 80% confluency.

### 2.2 Cell Viability Assay

Cells were cultured with nanoparticles or media conditioned with the nanoparticles (nanoparticles no longer present), with basal media as a control. To obtain the conditioned media, the particles were immersed in DMEM and incubated at 37  °C with shaking at 200 rpm for 24 h. After incubation, the particles were removed by centrifugation and filtration. RAW264.7 cells were seeded at a cell concentration of 5 × 10^4^ cells mL^−1^ in 96‐well plates (Nunc, Sigma–Aldrich). The effect of concentration of the monodispersed Si‐NPs (100 mol% SiO_2_), 4.4Sr‐BGNPs, 9.4Sr‐BGNP, and conditioned media on the in vitro metabolic activity of undifferentiated RAW264.7 cells was screened using a water‐soluble tetrazolium salt (WST‐1) assay. Si‐NPs were used as a control here to determine whether the presence of the vehicle silica particles caused a reduction of metabolic activity, relative to basal media, without the Sr present. After a 24 h attachment period in basal DMEM medium, the medium was replaced with media containing Si‐NPs, 4.4Sr‐BGNPs, 9.4Sr‐BGNPs or conditioned media at a particle concentration of 0, 50, 100, 150, 200, and 250 µg mL^−1^ for a 24 h (pulse), then the particles not associated to the cells were removed by washing with phosphate‐buffered saline (PBS) buffer twice. The concentrations were chosen in accordance with previous studies on bioactive nanoparticles.^[^ ^19^, ^34^ ^]^ After a further 1 day and 2 days in culture without adding further particles or conditioned media (chase), the cell metabolic activity was measured using a WST‐1 assay. Separately, undifferentiated RAW264.7 cells were exposed for 1 day and 2 days in culture to the conditioned media, as described previously.^[^ ^25^ ^]^

### 2.3 Osteoclast Differentiation

**Scheme 1:**
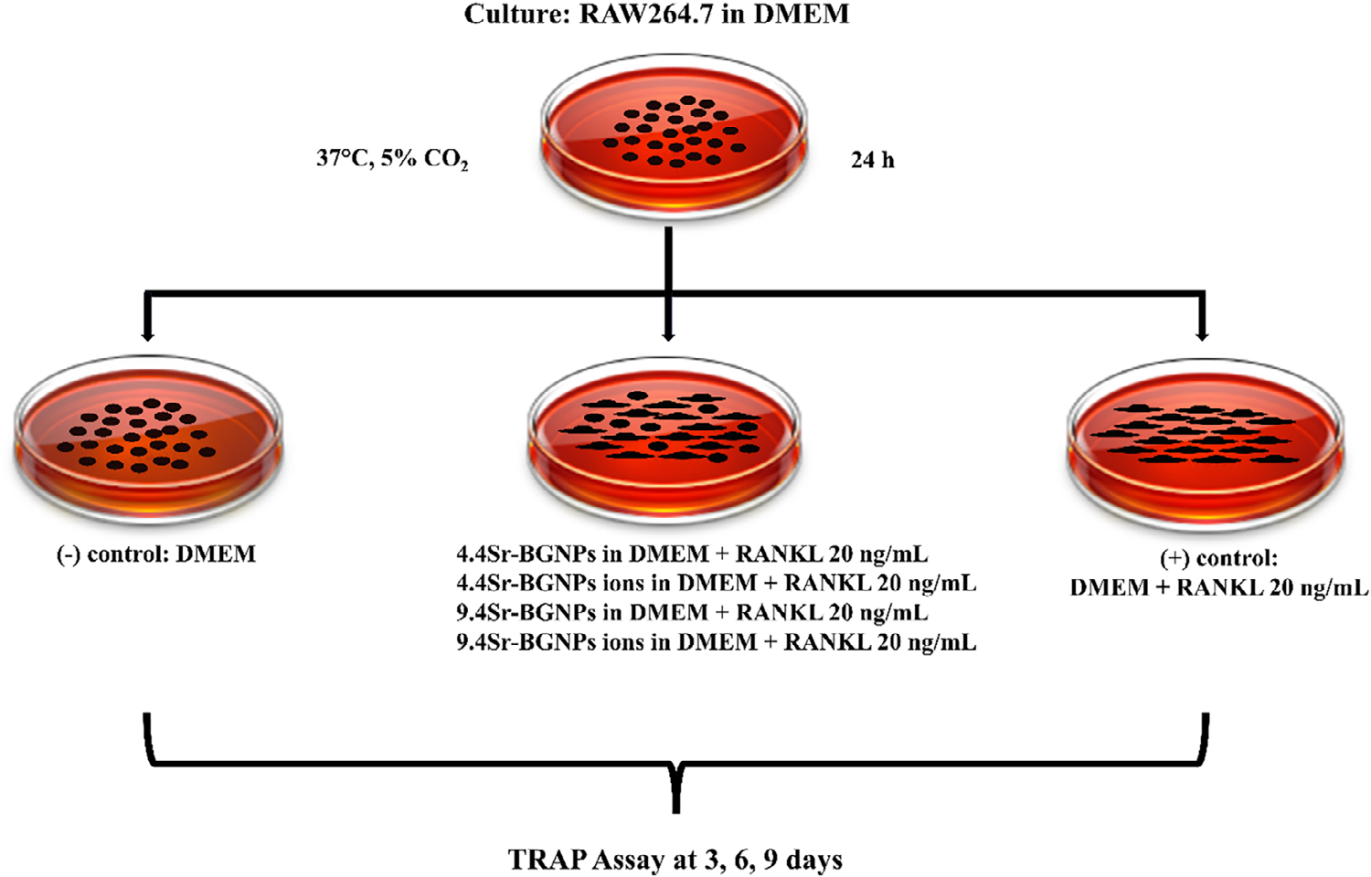
Method for investigating osteoclastogenic differentiation of RAW264.7 cells into multinucleated osteoclasts, with and without Sr‐BGNPs (containing 4.4 mol% or 9.4 mol% SrO) or media conditioned with Sr‐BGNPs (ions).

RAW264.7 cells were seeded at a concentration of 5 × 10^4^ cells mL^−1^ in 6‐well plates. To differentiate RAW264.7 cells to osteoclasts, the DMEM was supplemented with 20 ng mL^−1^ of soluble *murine* NF‐κB ligand (RANKL, PeproTech EC).^[^ ^35^ ^]^ The cells were then exposed to Sr‐BGNPs or conditioned media at a particle concentration of 250 µg mL^−1^ (+ RANKL), as illustrated in **Scheme** **1**. This concentration was chosen to maximize Sr loading without causing toxicity. Tartrate‐resistant acid phosphatase (TRAP) activity, a marker of osteoclast differentiation and resorptive activity, was measured after 3, 6, and 9 days in culture using an Acid Phosphatase Assay Kit (Colorimetric) (Abcam), following the manufacturer's instructions. To determine the formation of multinucleated osteoclasts, cells were washed with PBS and then fixed with 4% (w/v) paraformaldehyde in PBS for 15 min at room temperature after 9 days in culture. Cells were then washed (twice) with PBS and permeabilized with 1% (v/v) Triton X‐100 in PBS for 5 min before being stained with Alexa 568‐conjugated phalloidin (1:100, Molecular Probes). Cell nuclei were stained with 4',6‐diamidino‐2‐phenylindole, dihydrochloride (DAPI, Thermo Fisher Scientific). Samples were imaged using a Zeiss LSM‐510 inverted microscope.

### 2.4 Co‐Culture System

**Scheme 2:**
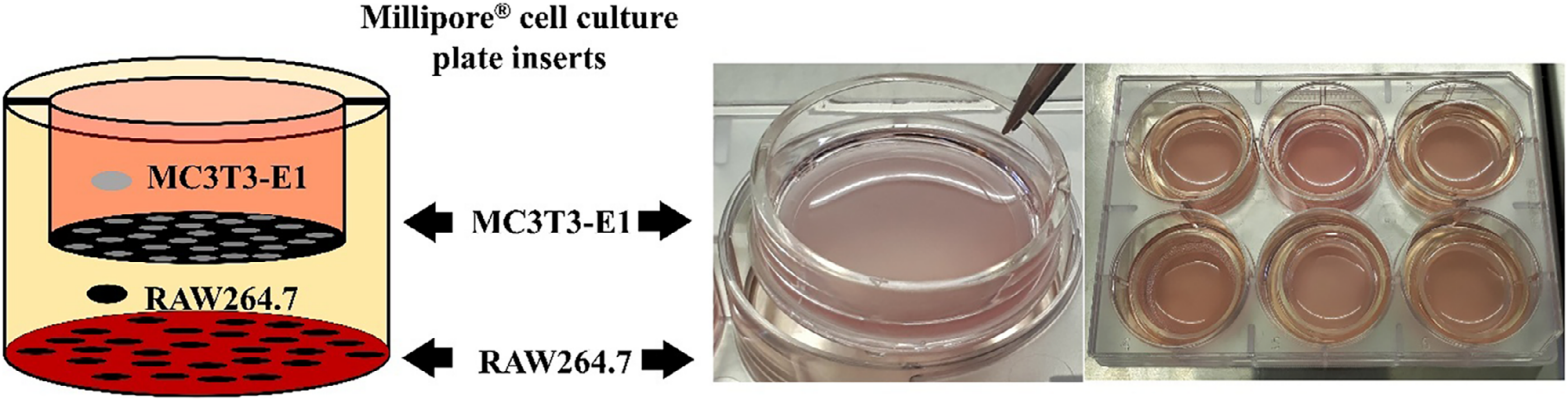
MC3T3‐E1‐RAW264.7 indirect co‐culture cell seeding.

Millipore Millicell cell culture plate inserts (Sigma–Aldrich) were used to set up a co‐culture system for the two types of cells: MC3T3‐E1 (*murine* osteoblast like cell) and RAW264.7 (mouse leukaemic monocyte macrophage cell line). RAW264.7 cells (at a concentration of 5 × 10^4^ cells mL^−1^) were seeded in the 6‐well plates (Nunc, Sigma–Aldrich) and MC3T3‐E1 cells (at a concentration of 5 × 10^4^ cells mL^−1^) were placed in the 6‐well inserts with 0.4 µm pore size (Millipore, Sigma–Aldrich). The indirect co‐culture system (**Scheme** **2**) was cultured in α‐MEM supplemented with 10% (v/v) foetal bovine serum (FBS), 100 U mL^−1^ penicillin and 100 µg mL^−1^ streptomycin (Gibco; Thermo Fisher Scientific), at 37 °C, 5% CO_2_ and fully humidified atmosphere for 24 h to allow the cells to form a monolayer.

**Scheme 3:**
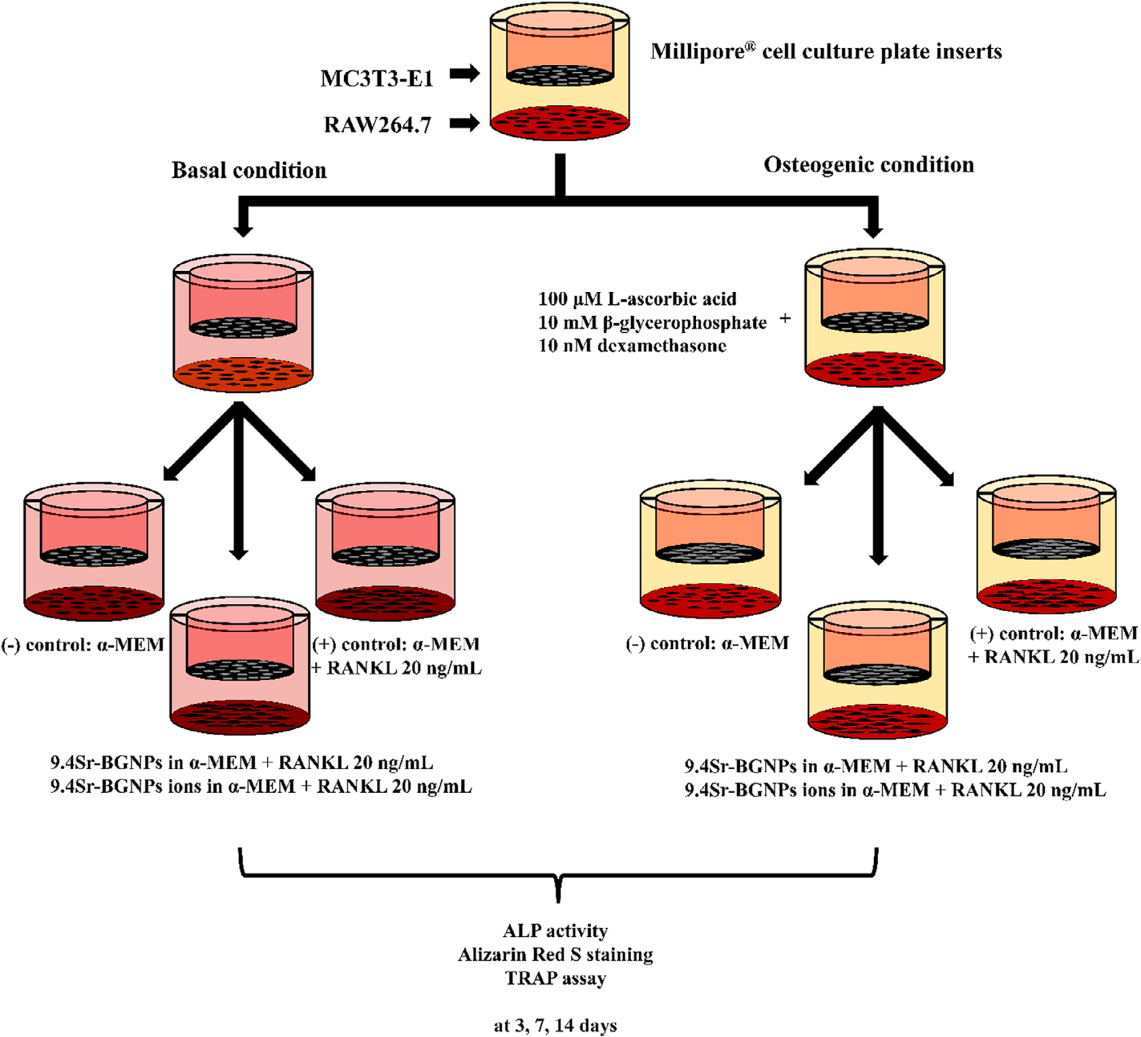
Investigating the effect of Sr‐BGNPs and their dissolution ions on MC3T3‐E1‐RAW264.7 indirect co‐culture.

The co‐culture system was cultured under basal and osteogenic (basal supplemented with 100 µM L‐ascorbic acid, 10 mM β‐glycerophosphate, and 10 nM dexamethasone) conditions, with and without 20 ng mL ^−1^ of RANKL. Cells were exposed to Sr‐BGNPs or media conditioned with 9.4Sr‐BGNPs dissolution products, using a particle concentration of 250 µg mL^−1^, both in the basal and osteogenic conditions (basal media supplemented with 100 µM L‐ascorbic acid, 10 mM β‐glycerophosphate, and 10 nM dexamethasone (DEX)^[^ ^25^ ^]^ (**Scheme** **3**). The culture media was changed twice a week to ensure a high nutrient concentration. The Sr‐BGNPs and conditioned media were incubated with the cells after every medium change.

At time intervals of up to 14 days, the ALP activity of treated MC3T3‐E1 cells was measured using the Alkaline Phosphatase (ALP) Assay Kit (Colorimetric) (Abcam) according to the manufacturer's instructions. Cells were also fixed with 4% paraformaldehyde in PBS. Cells were stained with 2% Alizarin Red S in PBS at pH 4.2 to detect calcified tissue formation. For osteoclast differentiation, the TRAP histochemical stain was used (378‐ A) to identify a marker of resorbing activity, according to the manufacturer's instructions, and RANKL levels were quantified using the RANKL (TNFSF11) Mouse ELISA Kit (ab100749, Abcam) in accordance with the manufacturer's instructions following 3, 7, and 14 days in culture.

### 2.5 Statistical Analysis

Statistical analyses were performed by one‐way analysis of variance (ANOVA) in Minitab with the appropriate post hoc comparison test (Tukey's test). A *p‐ value* <0.05 was considered significant. The graphs shown present the results as the mean value with the standard deviation (SD) as the error bars.

## 3 Results and Discussion

### 3.1 Monoculture

**Figure 1:**
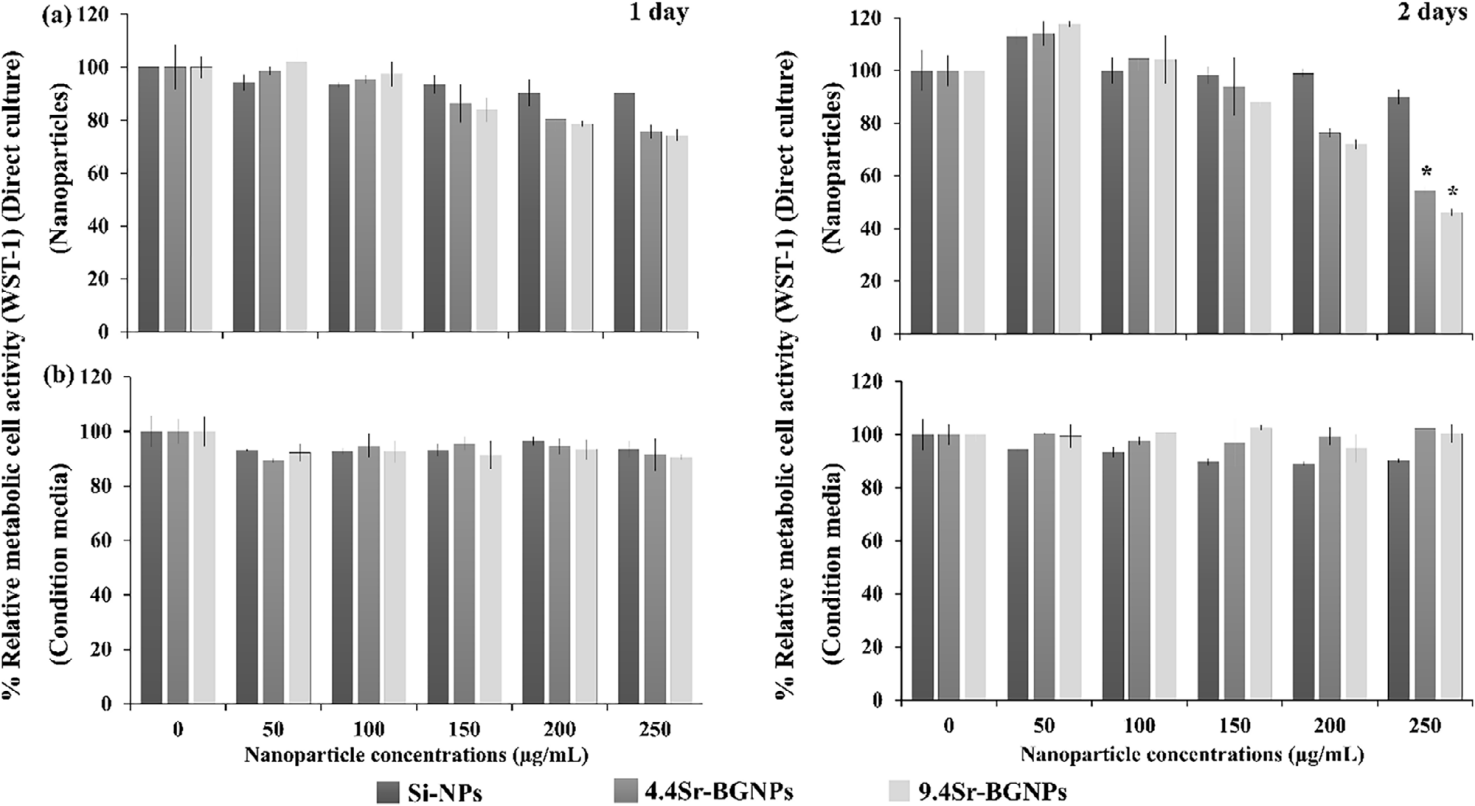
Cell viability of RAW264.7 cells (WST‐1 assay) after 1 day (left) and 2 days (right) of exposure to a) Si‐NPs, 4.4Sr‐BGNPs, and 9.4Sr‐BGNPs at a range of concentrations b) media conditioned by ions released from the particles. Data were normalized with respect to the positive control (cells cultured in nanoparticle‐free medium), expressed as mean ± SD of three independent experiments (n = 6). (^,^) indicates a statistically significant difference compared to controls (*p < 0.05*).

The relative cell viability of RAW264.7 cells when exposed to Sr‐BGNPs (particles in direct contact with the cells) at concentrations of 0, 50, 100, 150, 200, and 250 µg mL^−1^ for 1 and 2 days in culture, relative to the positive control (viability of cells cultured in nanoparticle‐free medium), is represented in **Figure** **1**a. Figure 1b shows the results for the cells exposed only to the media conditioned with the dissolution ions of the BGNPs, prepared in media at the same concentrations. The cell viability of RAW264.7 cells exposed to the Sr‐BGNPs was both dose and time‐dependent: a high concentration of 250 µg mL^−1^ caused a statistically significant reduction in metabolic activity of cells exposed to 4.4Sr‐BGNPs and 9.4Sr‐BGNPs (*p<0.05*) after 2 days, indicating a decrease in metabolic activity relative to the control. Previous work reported that Si‐NPs induced reactive oxygen species (ROS) formation that caused toxicity to RAW 264.7 cells,^[^ ^36^, ^37^ ^]^ but the Si‐NPs here did not cause any drop in viability. Figure 1 therefore shows that neither the vehicle Si‐NPs nor the nanoparticles containing up to 9.4 mol% Sr caused a reduction of metabolic activity until a concentration of 250 µg mL^−1^. Our previous study reported that 4.4Sr‐BGNPs and 9.4Sr‐BGNPs at a concentration of 250 µg mL^−1^ promoted osteoblastic differentiation of human mesenchymal stem cells (hMSCs)^[^ ^26^ ^]^ and exhibited anti‐inflammatory effects by inducing M2 macrophage polarization.^[^ ^38^ ^]^ Here, although this concentration reduced the metabolic activity of treated cells, it did not induce cell death, as confirmed by nuclear staining (Figure 3). Therefore, 4.4Sr‐BGNPs and 9.4Sr‐BGNPs at a particle concentration of 250 µg mL^−1^ were used in the subsequent experiments of this study to maximise Sr loading without causing toxicity.

**Figure 2:**
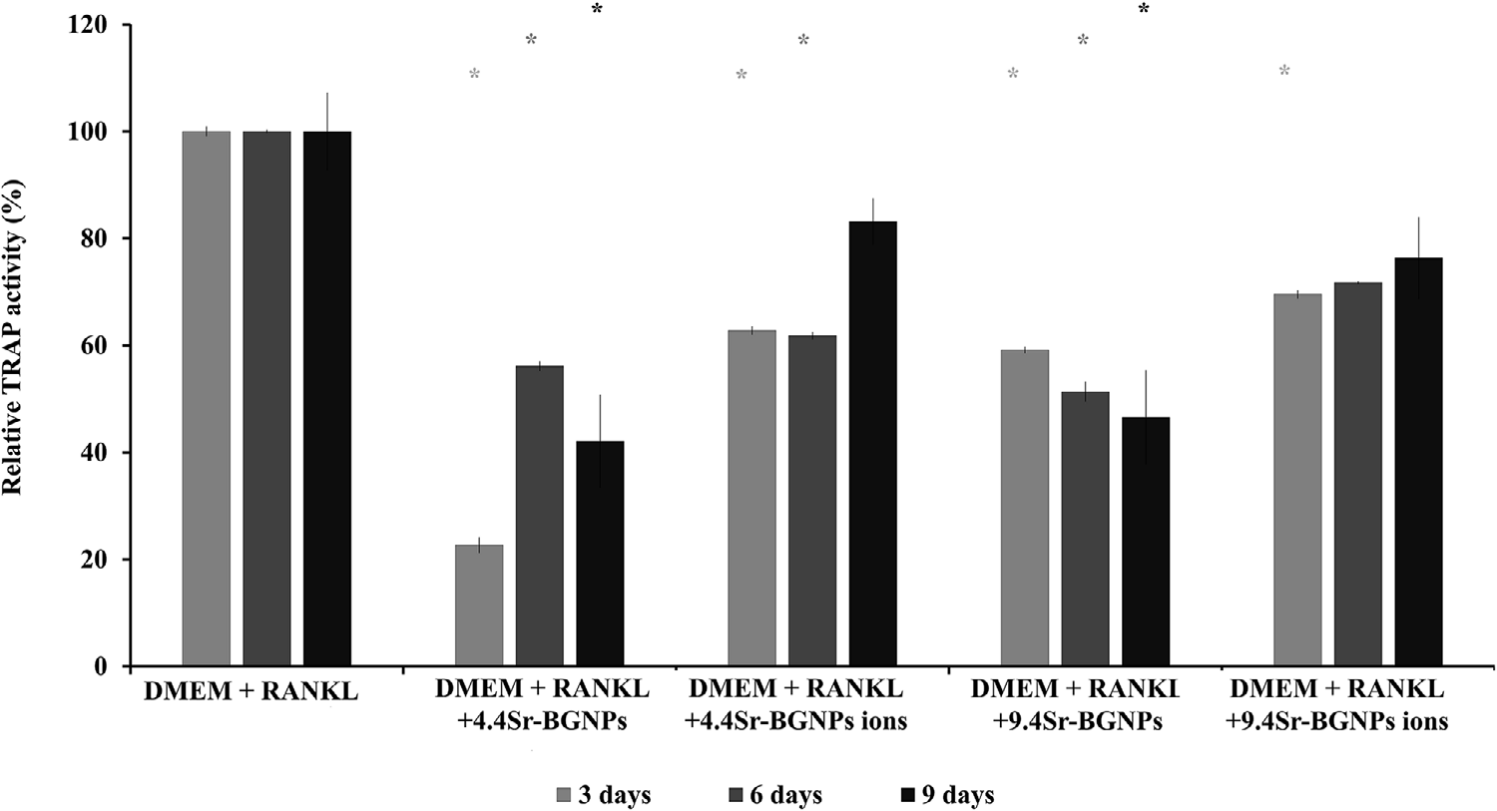
Relative TRAP activity (%) of RAW264.7 cells following culture with Sr‐BGNPs or their conditioned media (the particle concentration at 250 µg mL^−1^) in DMEM supplemented with 20 ng mL^−1^ RANKL for 3, 6, and 9 days. The *p*‐values of *p<0.05* are considered statistically significant and are denoted with ^,^, compared to DMEM+RANKL without particles.

Osteoclasts play a key role in bone resorption. Whether RAW264.7 cells differentiated into osteoclasts was investigated through the detection of TRAP activity and the formation of multinucleated osteoclasts in the mono‐culture system. The TRAP activity significantly decreased in RAW264.7 cells after treatment with 4.4Sr‐BGNPs and 9.4Sr‐BGNPs (the particle concentration at 250 µg mL^−1^) following 3, 6, and 9 days in culture (**Figure** **2**), relative to cells cultured in DMEM with RANKL. TRAP served as a functional enzymatic marker for osteoclast activity. The observed reduction in metabolic activity was evidenced by decreased TRAP levels, indicating that direct exposure to particles affected osteoclast metabolic function. Consistent with previous reports, low concentrations of Sr more effectively inhibited osteoclastogenesis and TRAP expression during the first five days compared to higher Sr concentrations.^[^ ^39^ ^]^ The effect of the dissolution ions, containing 6–9 mg L^−1^ of Sr (**Table** **2**), without the particles, was less pronounced, with the TRAP activity of RAW264.7 cells decreasing after 3 and 6 days in culture, but there was no significant reduction after 9 days in culture. Larger Sr‐substituted BG particles (400‐600 nm, containing ≈6 mol% SrO_2_) were previously found to suppress osteoclastogenesis of RAW264.7 cells,^[^ ^40^, ^41^ ^]^ with little effect of changing concentrations.

**Table 2:** Elemental content of conditioned media following soaking of Sr‐BGNPs (the particle concentration at 250 µg mL^−1^) in DMEM media, measured by ICP‐OES.

	Ion concentrations [mg L^−1^]			
S	[Si]	[Ca]	[P]	[Sr]
DMEM	0 ± 0	68.39 ± 0. 47	32.40 ± 0.62	0 ± 0
DMEM+RANKL	0 ± 0	69.95 ± 0.53	32.60 ± 0.45	0 ± 0
DMEM+RANKL +4.4Sr‐BGNPs Ions	47.74 ± 2.05	62.22 ± 4.46	22.91 ± 5.91	5.56 ± 0.95
DMEM+RANKL +9.4Sr‐BGNPs Ions	43.00 ± 0.34	60.71 ± 3.17	25.32 ± 3.08	9.05 ± 1.42

**Figure 3:**
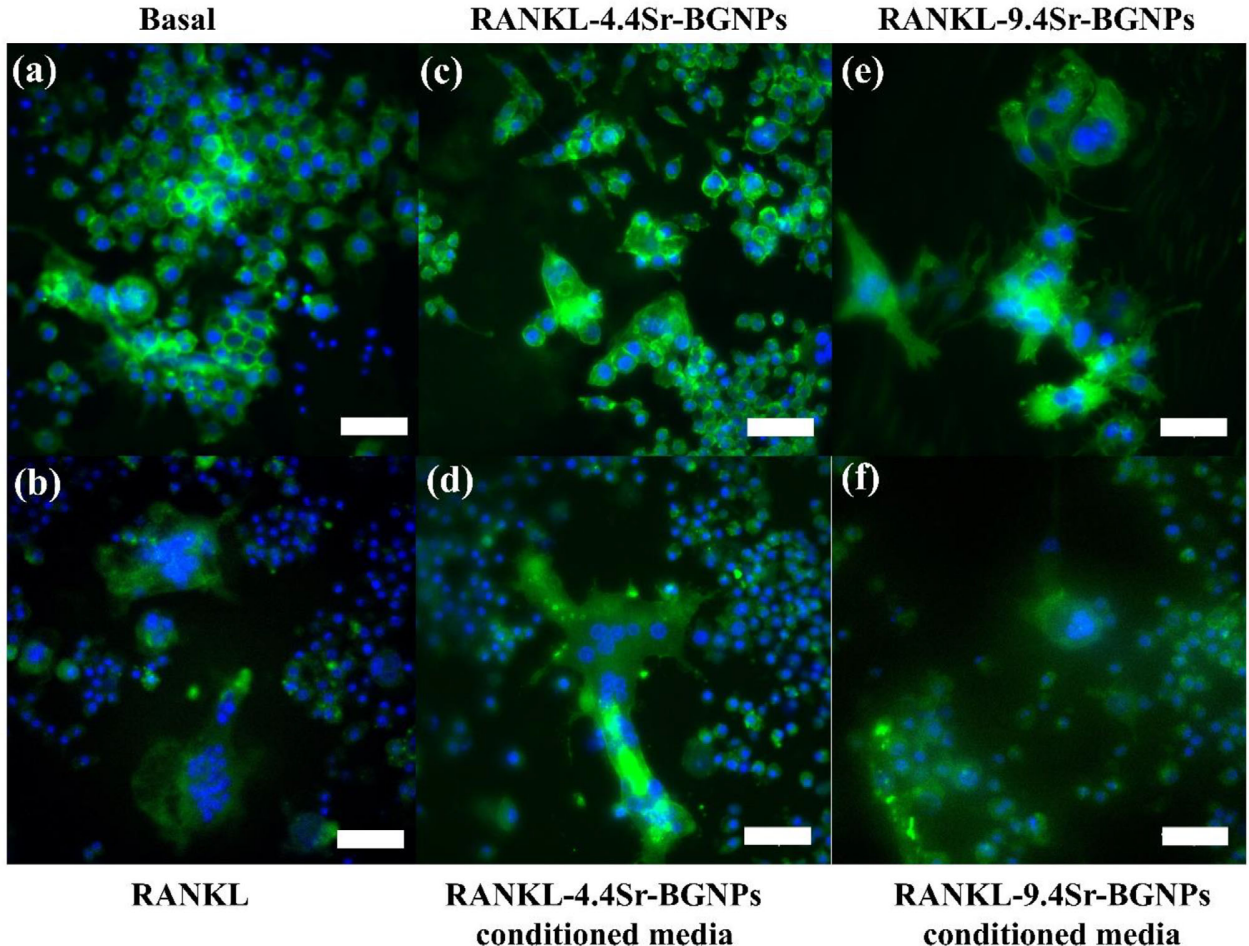
Fluorescence microscopy images of cells after 9 days in mono‐culture a) RAW264.7 cells in basal media; b) RANKL‐stimulated RAW264.7 cells; c) RANKL‐stimulated RAW264.7 cells exposed to 4.4Sr‐BGNPs; d) RANKL‐stimulated RAW264.7 cells exposed to 4.4Sr‐BGNPs ions (conditioned media); e) RANKL‐stimulated RAW264.7 cells exposed to 9.4Sr‐BGNPs, and f) RANKL‐stimulated RAW264.7 cells exposed to 9.4Sr‐BGNPs ions (conditioned media). Sr‐BGNP concentration was 250 µg mL^−1^. Nucleus (blue) and cytoplasm (green). Scale bar is 150 µm.

The transformation of mononucleated osteoclasts into multinucleated osteoclasts through cell‐cell fusion is a critical step in identifying osteoclast differentiation.^[^ ^42^ ^]^ To determine the influence of Sr‐BGNPs on the formation of multinucleated osteoclasts, the morphology of RANKL‐stimulated RAW264.7 cells was monitored by fluorescent microscopy (**Figure** **3**). After 9 days in culture, in the absence of RANKL, multinucleated osteoclasts did not form in the negative control (Figure 3a) of basal media, while in the presence of RANKL (the positive control), multinucleated RANKL‐stimulated RAW264.7 cells formed via cell fusion (Figure 3b). Interestingly, the multinucleated RANKL‐stimulated RAW264.7 cells exposed to Sr‐BGNPs and conditioned media (the particle concentration at 250 µg mL^−1^) were less able to form (Figure 3c–f) than those not exposed to Sr‐BGNPS, indicating their involvement in inhibiting osteoclastogenesis. These results are consistent with previous studies that reported that Sr‐substituted BG inhibited osteoclastogenesis.^[^ ^35^, ^41^, ^43^, ^44^ ^]^ In monoculture, Sr‐BGNPs have been shown to stimulate osteoblast activities^[^ ^25^, ^26^ ^]^ and inhibit osteoclast activities, including TRAP activity, and the formation of multinucleated osteoclasts. The results show that Sr‐BGNPs and their dissolution products reduce the formation of osteoclasts in a monoculture system. However, bone formation and bone remodeling are regulated by cross‐communication between osteoblasts and osteoclasts. To test whether the Sr‐BGNPs induce bone formation by stimulating osteoblast and inhibiting osteoclast activities, the indirect osteoblast and osteoclast co‐culture system was set up.

### 3.2 Co‐Culture

**Figure 4:**
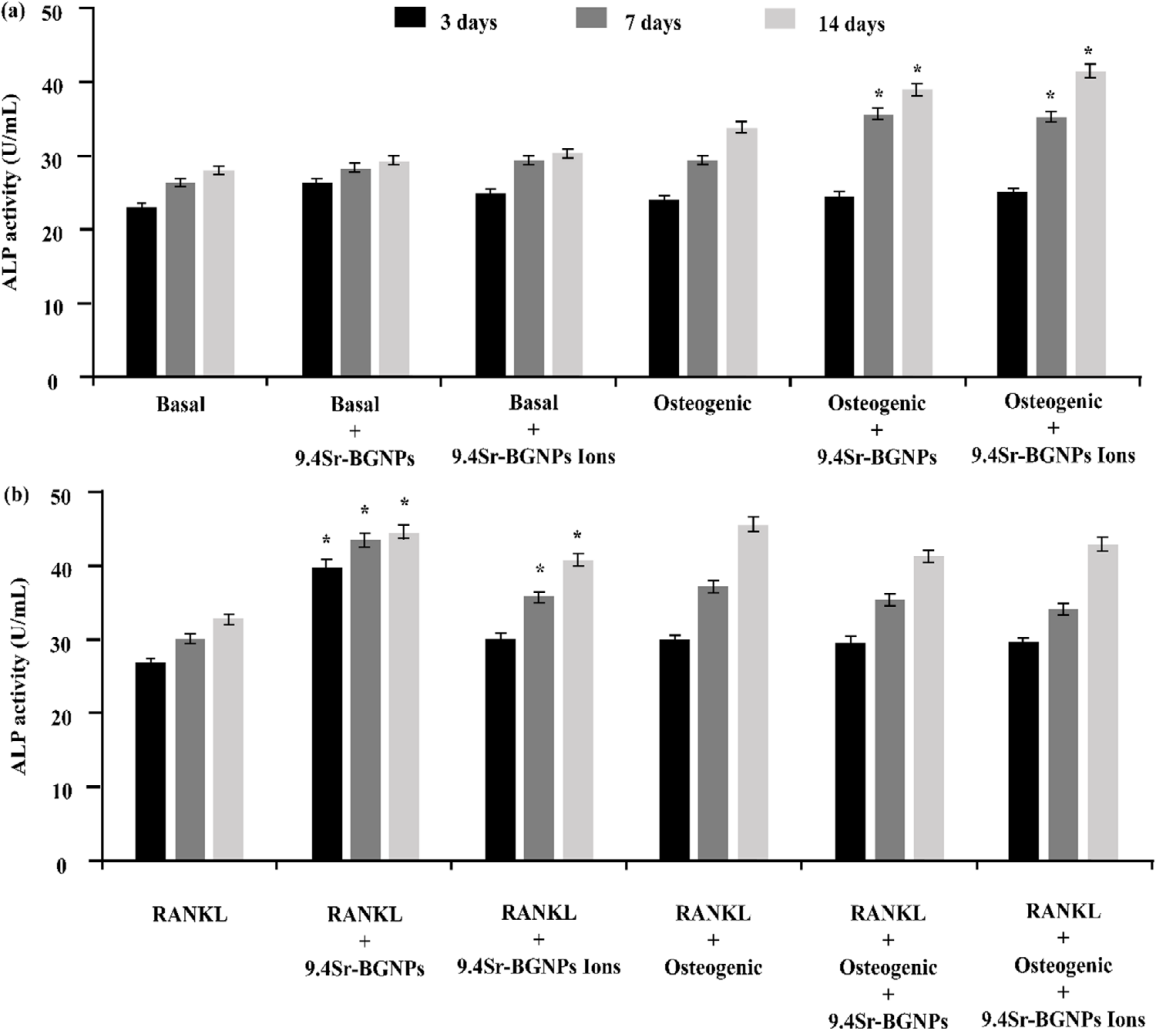
ALP activity of MC3T3‐E1 cells in co‐culture with RAW264.7 cells 3, 7, and 14 days after MC3T3‐E1s had been treated with 9.4Sr‐BGNPs, or their conditioned media, in basal and osteogenic conditions in a) absence of RANKL, and b) presence RANKL concentration at 20 ng mL^−1^ (n = 3) (^,^ *P<0.05*). Particle concentration was 250 µg/mL.

The 9.4Sr‐BGNPs were selected for co‐culture experiments due to their higher strontium ion release (9.1 mg L^−1^) compared to the 4.4Sr‐BGNPs (5.6 mg L^−1^), while demonstrating a comparable inhibitory effect on osteoclast differentiation. Moreover, our previous study showed that the 9.4Sr‐BGNPs significantly enhanced osteoblast differentiation, further justifying their use in the co‐culture model.^[^ ^26^ ^]^ The effect of culture of 9.4Sr‐BGNPs or their conditioned media (at a particle concentration of 250 µg mL^−1^) on an in vitro co‐culture system for osteoclasts and osteoblasts (MC3T3‐E1 and RAW264.7, Scheme 2) was investigated using the Millipore insert co‐culture system, with and without osteogenic and osteoclastic supplementation (Scheme 3). The differentiation of MC3T3‐E1 into osteoblasts was detected through the ALP activity assay and Alizarin Red S staining. ALP, an early marker of osteoblast differentiation, is involved in the initial differentiation step to form the nucleation of the mineralization process within the matrix vesicles, while Alizarin Red S staining identifies mineralized nodules.^[^ ^32^, ^45^ ^]^ In the absence of RANKL supplement (**Figure** **4**a) and osteogenic supplements, little change in ALP activity was observed, even with Sr‐BGNPs present. When osteogenic supplements were used, ALP activity of MC3T3‐E1 cells treated with 9.4Sr‐BGNPs or their conditioned media increased significantly (*p < 0.05*) after 7 and 14 days in culture, compared to the osteogenic control (Figure 4a). Osteogenic supplements alone did not cause a significant increase, implying that the combination of the osteogenic supplements with Sr‐BGNPs or their dissolution products acts synergistically to stimulate ALP activity. With RANKL supplementation, the ALP activity of the MCT3T‐E1 cells treated with 9.4Sr‐BGNPs and their dissolution products after 3, 7, and 14 days (under the basal condition) increased significantly compared to the control (Figure 4b). These results are consistent with previous studies reporting that Sr‐BGNPs and dissolution ions enhanced the ALP activity (qualitatively) of MCT3T‐E1 in monoculture after 21 days, with particles or dissolution ions reintroduced with every media change, every three days.^[^ ^25^ ^]^ These results suggested that Sr release from the 9.4Sr‐BGNPs can synergistically enhance osteoblastic differentiation pathways that are already stimulated by either osteogenic supplements or RANKL. This is consistent with previous reports indicating that strontium ions can stimulate osteoblast differentiation while also modulating RANKL‐mediated pathways.^[^ ^46^ ^]^ However, when osteogenic supplements, RANKL, and 9.4Sr‐BGNPs were combined, the enhancement of ALP activity was not observed. We hypothesized that this might be due to signaling crosstalk and pathway saturation. RANKL activates osteoclastogenesis‐related signaling, such as NF‐κB and NFATc1,^[^ ^47^ ^]^ which counterbalance the osteogenic signaling promoted by osteogenic supplements and 9.4Sr‐BGNPs. The simultaneous stimulation of both osteogenic and osteoclastogenic pathways could therefore reduce ALP activity. In addition, the observed decrease in mineralisation when RANKL was combined with 9.4Sr‐BGNPs may be attributed to the fact that RANKL‐induced osteoclastogenic signaling reduces mineral deposition, even though ALP activity, an early marker of differentiation, was increased.^[^ ^48^, ^49^, ^50^ ^]^ In summary, ALP upregulation reflects early differentiation, but RANKL‐driven resorption signaling limits the later stage of matrix mineralisation.

**Figure 5:**
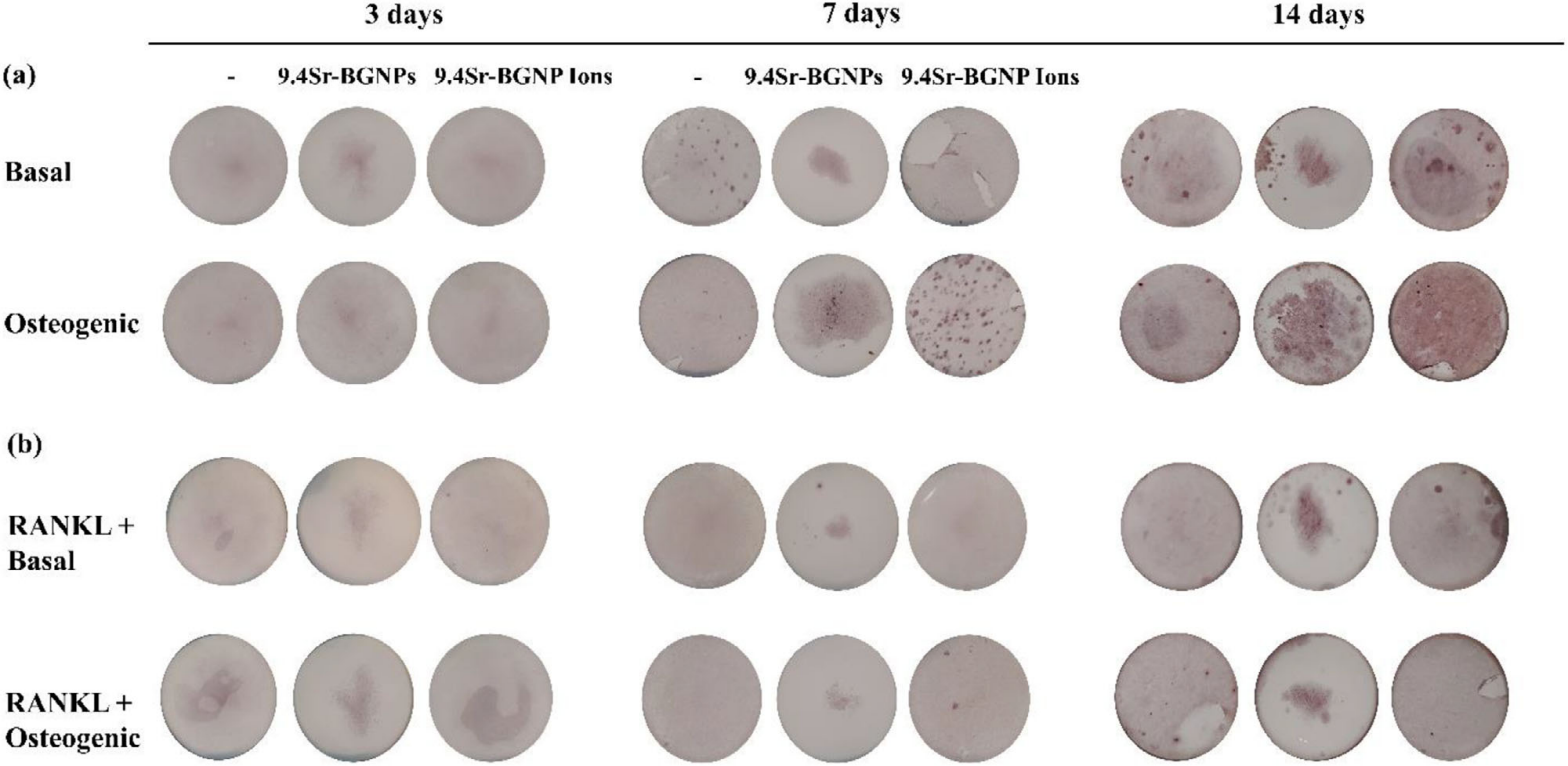
The effect of culture of 9.4Sr‐BGNPs and their conditioned media on MC3T3‐E1 mineralization in vitro in the indirect co‐culture with RAW264.7 a) without RANKL and b) with RANKL. MC3T3‐E1 cells were indirectly co‐cultured on a Millipore insert co‐culture for 3, 7, and 14 days. Particle concentration was 250 µg mL^−1^.

Positive Alizarin Red S staining is an indication of calcium deposition and nodule formation. In the absence of RANKL supplement, mineralized nodule formation was detected in MC3T3‐E1 in the co‐culture with RAW264.7 treated with 9.4Sr‐BGNPs and their conditioned media after 14 days in culture under the basal condition and after 7 days when also cultured with osteogenic supplements (**Figure** **5**a).  This indicates that 9.4Sr‐BGNPs and their dissolution products stimulated mineralisation of MC3T3‐E1 in the indirect co‐culture system with and without osteogenic supplements. In the presence of RANKL supplement, mineralization was much less that it was when no RANKL was added; no significant calcium deposition and nodule formation were observed in both the basal and osteogenic conditions (Figure 5b), implying that the RANKL supplement could suppress nodule formation of MC3T3‐E1 in the co‐culture system due to the greater osteoclast activity resorbing the mineral.^[^ ^51^, ^52^ ^]^

**Figure 6:**
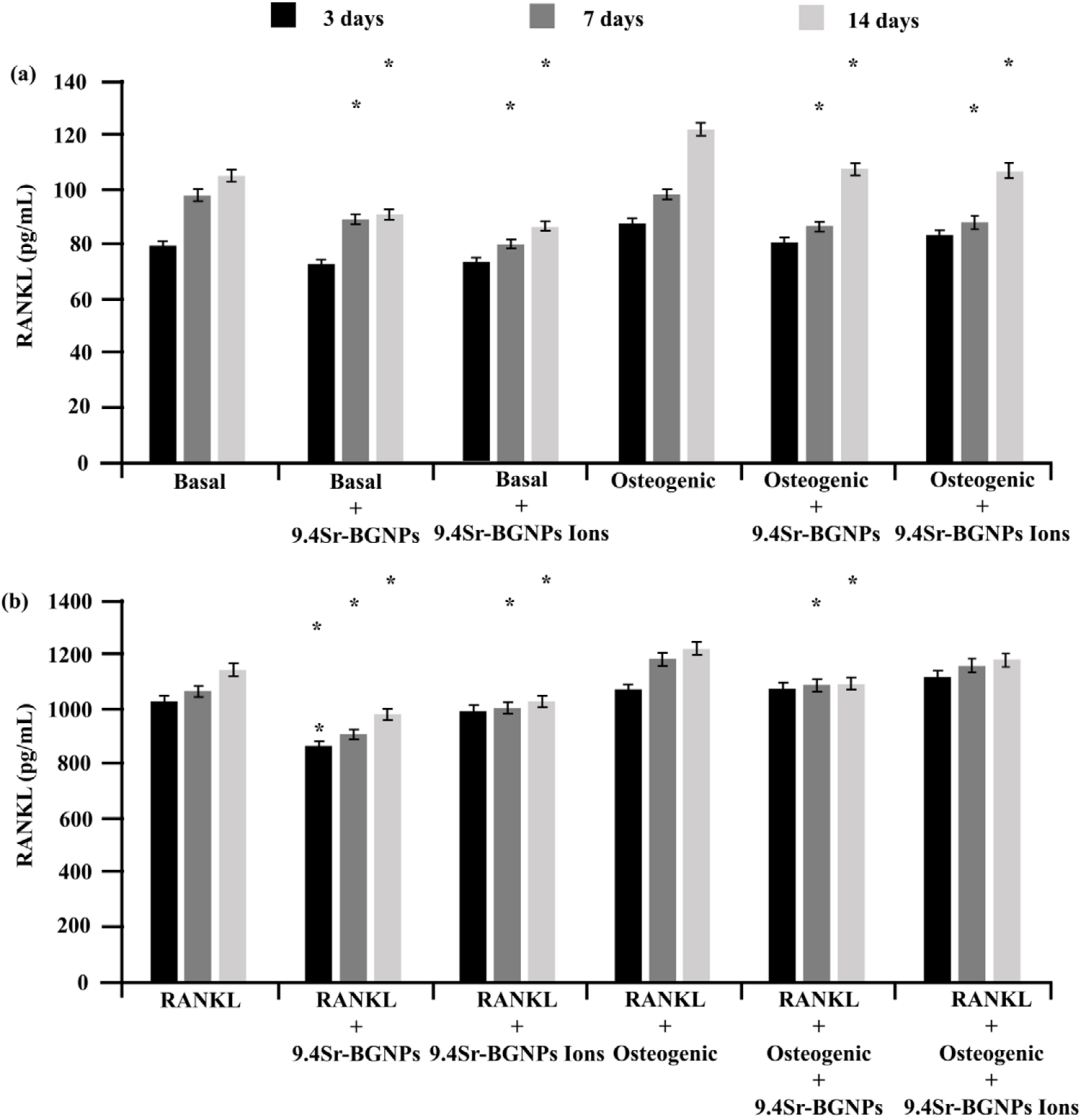
Total RANKL secretion was evaluated by ELISA following 3, 7, and 14 days’ treatment with 9.4Sr‐BGNPs and their dissolution ions at a particle concentration of 250 µg mL^−1^ in the basal and osteogenic conditions a) without RANKL supplementation, b) with RANKL supplementation at 20 ng mL^−1^ (n = 3). The *p‐values* of *p<0.05* are considered statistically significant and are denoted with ^,^, compared to the control without particles and dissolution ions.

The communication between osteoclast and osteoblast through cell‐cell contact is critical in bone remodeling, with osteoblasts regulating osteoclastogenesis through the OPG/RANKL system. RANKL, a key regulator of osteoclastogenesis, secreted by osteoblasts, binds to the RANK receptor, resulting in the promotion of osteoclast formation.^[^ ^6^, ^7^ ^]^ Therefore, the level of RANKL secreted from MC3T3‐E1 in the indirect co‐culture system with RAW264.7 was investigated. The statistically significant decrease in RANKL expression was observed in MC3T3‐E1 cells exposed to 9.4Sr‐BGNPs or conditioned media, compared to the control, under both basal and osteogenic conditions. This reduction was evident in the absence (**Figure** **6**a) and presence (Figure 6b) of RANKL supplementation at both 7 and 14 days. This implies that 9.4Sr‐BGNPs and their dissolution products inhibit osteoclast formation via the suppression of RANKL secretion levels from osteoblastic cells.

**Figure 7:**
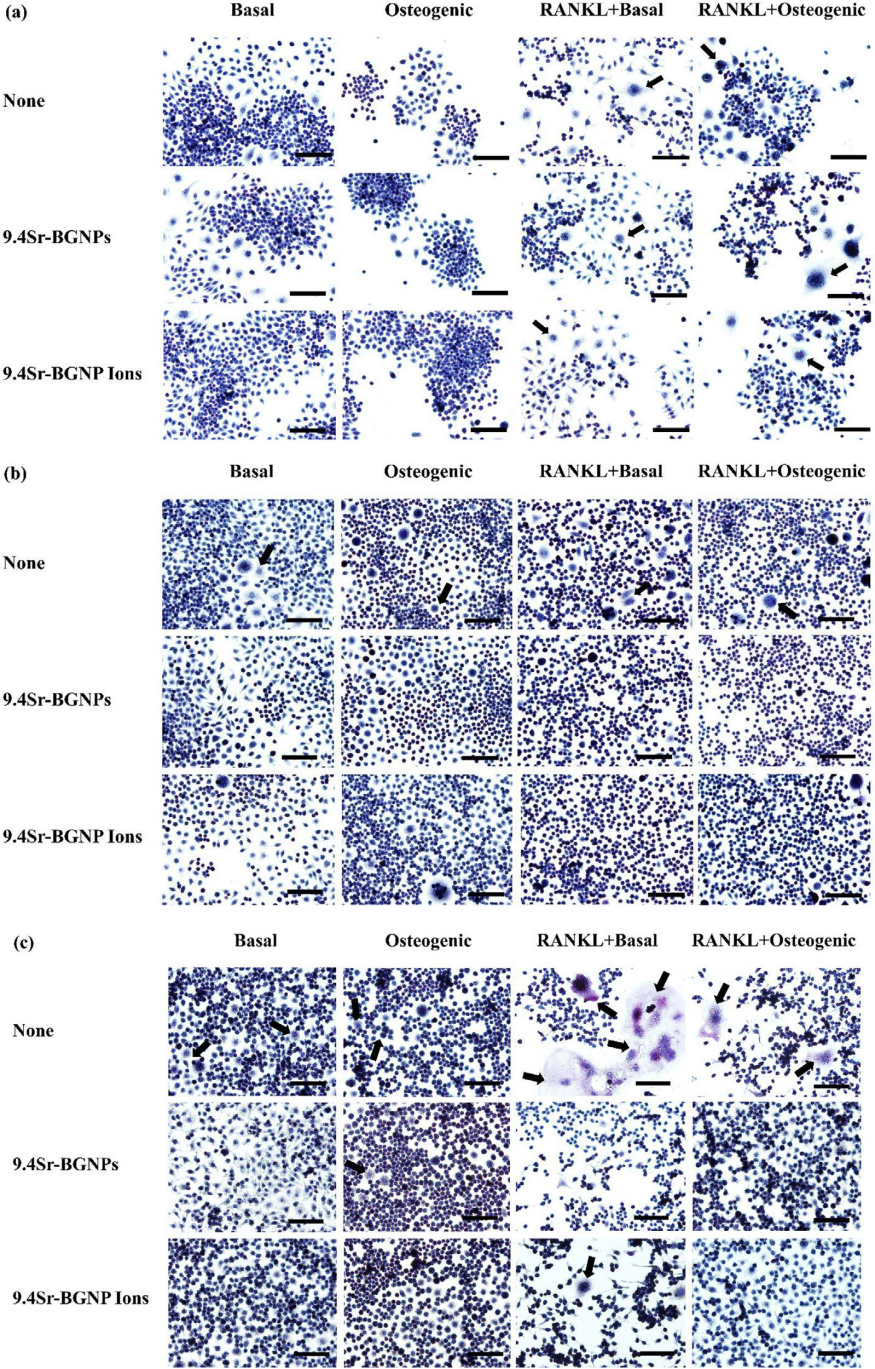
TRAP staining of RAW264.7 cells in co‐culture treated with 9.4Sr‐BGNPs or media conditioned with 9.4Sr‐BGNPs dissolution ions (particle concentration at 250 µg mL^−1^) under basal and osteogenic conditions in the presence and in the absence of RANKL (20 ng mL^−1^) for a) 3, b) 7, and c) 14 days. Scale bars represent 100 µm. Positive staining cells are represented in purple colour with multinucleation. The black arrow indicated multinucleation.

To understand the effect of RANKL secreted from osteoblasts on osteoclastogenesis, TRAP activity staining, a marker for the early osteoclast differentiation, was used to examine the differentiation of RAW264.7 into multinucleated osteoclasts in the indirect co‐culture system with MC3T3‐E1. RANKL was previously reported to promote TRAP activity within 3 days.^[^ ^53^, ^54^ ^]^  Thus, in this study, TRAP activity staining of RAW264.7 exposed to 9.4Sr‐BGNPs and their dissolution ions, with and without RANKL, was investigated following 3, 7, and 14 days of co‐culture. Without RANKL supplements, no multinucleated cell formation was detected after 3 days for both under basal and osteogenic control groups (**Figure** **7**a), while some multinucleated cells were observed in the controls in both under basal and osteogenic conditions after 7 (Figure 7b and 14 days (Figure 7c). The number of multinucleated cells was reduced when either Sr‐BGNPs or their dissolution products were added at 7 and 14 days. In the presence of RANKL, some multinucleated cells were stained positively for TRAP (purple) after 3 days (Figure 7a) in all conditions. The number of TRAP multinucleated treated cells decreased after 7 (Figure 7b) and 14 days (Figure 7c) when exposed to Sr‐BGNPs and their dissolution products. The TRAP‐positive cells (purple with multi‐nucleus aggregation) were considered to be osteoclasts, and their numbers were counted and relatively compared to the control groups: basal, osteogenic, RANKL+basal, and RANKL+osteogenic, respectively.

**Figure 8:**
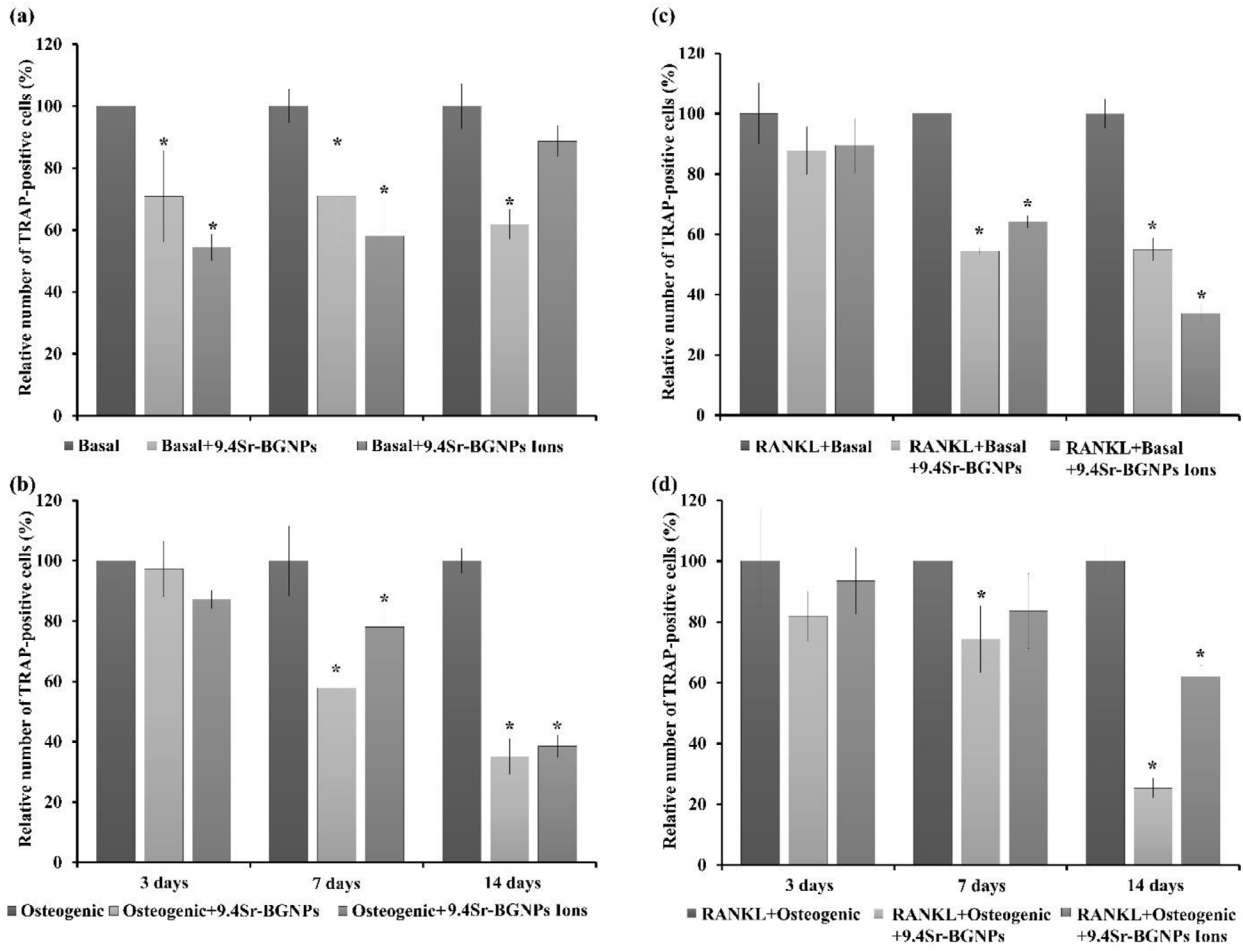
Quantitative results of the relative number of TRAP‐positive cells to those in control in co‐culture under a) basal condition, b) osteogenic condition, c) basal media supplemented with RANKL, and d) osteogenic media supplemented with RANKL. In each condition, the cells were exposed to 9.4Sr‐BGNPs or media conditioned with 9.4Sr‐BGNPs dissolution ions (particle concentration at 250 µg mL^−1^). Results are expressed as the mean ± SD compared to the non‐supplemented control (n = 4 staining areas) (^,^ *P < 0.05*).

To quantify the number of TRAP‐ positive multinucleated osteoclast differentiation in RAW264.7 cells, cultured in the indirect co‐culture system, the number of TRAP‐ positive cells was counted and compared to the control groups; basal (**Figure** **8**a), osteogenic (Figure 8b), RANKL+basal (Figure 8c), and RANKL+osteogenic (Figure 8d).

There was a statistically significant reduction (*P < 0.05*) in the number of TRAP‐positive cells for cells exposed to Sr‐BGNPs or their dissolution ions after 7 and 14 days (Figure 8) under all conditions. A similar reduction was seen at 3 days under the basal condition. This means Sr‐BGNPs and ions released from the particles have a capacity to inhibit osteoclast differentiation through suppression of TRAP activity. Sr‐BGNPs and their dissolution ions reduced the early state differentiation of RAW 264.7 cells, which is consistent with previous reports on the effect of Sr on inhibiting osteoclasts in vitro.^[^ ^32^, ^55^ ^]^ These results indicated that the 9.4Sr‐BGNPs and ions released from the particles have an inhibitory activity on osteoclast differentiation by suppressing TRAP activity. Taken together, these results support our hypothesis that Sr‐BGNPs reduce bone resorption by inhibiting osteoclast activity.

## 4 Conclusion

Sr‐BGNPs and ions released from the Sr‐BGNPs enhance osteoblast differentiation and reduce osteoclast differentiation in vitro, both in monoculture and indirect co‐culture with RAW264.7 cells. The RAW264.7 cells differentiated into osteoclasts without supplementation with RANKL, according to TRAP staining. In monoculture, Sr‐BGNPs and released ions that were not toxic to RAW264.7 cells reduced TRAP activity and reduced the number of multinucleated osteoclasts, even when the media was supplemented with RANKL. The results from monoculture indicated that the differentiation potential of pre‐osteoclast‐like cells into osteoclast‐like cells was reduced when RAW264.7 cells were treated with Sr‐BGNPs and released ions in vitro. In the indirect osteoblast‐osteoclast (MC3T3‐E1‐RAW264.7) co‐culture system, the osteoblastic ALP activity and mineralized matrix formation of MC3T3‐E1 cells exposed to Sr‐BGNPs and their dissolution ions increased. The level of RANKL secreted from MC3T3‐E1 in the indirect co‐culture system with RAW264.7 was reduced when the cells were exposed to Sr‐BGNPs and their dissolution ions. Osteoclastogenesis was inhibited in the conditioned medium of osteoclast precursor‐like cells via the reduction in TRAP activity and multinucleated osteoclast formation. The osteoblasts produced RANKL but the RANKL secretion of MC3T3‐E1 cells statistically significantly decreased when cultured with the Sr‐BGNPs, resulting in a reduction of multinucleated osteoclasts. Sr‐BGNPs have great potential for osteoporosis treatment as it can reduce osteoclastogenesis while promoting osteogenesis. The co‐culture models should also be used in future testing of other bone remodeling‐based therapies.

## Conflict of Interest

The authors declare no conflict of interest.

## Data Availability

The data that support the findings of this study are available from the corresponding author upon reasonable request.
